# Advances in the development of antivirals for rotavirus infection

**DOI:** 10.3389/fimmu.2023.1041149

**Published:** 2023-03-17

**Authors:** Lin Jiang, Ao Tang, Lihua Song, Yigang Tong, Huahao Fan

**Affiliations:** College of Life Science and Technology, Beijing University of Chemical Technology, Beijing, China

**Keywords:** rotavirus, diarrhoea, Chinese medicine, natural compounds, viral replication cycle, immunotherapy

## Abstract

Rotavirus (RV) causes 200,000 deaths per year and imposes a serious burden to public health and livestock farming worldwide. Currently, rehydration (oral and intravenous) remains the main strategy for the treatment of rotavirus gastroenteritis (RVGE), and no specific drugs are available. This review discusses the viral replication cycle in detail and outlines possible therapeutic approaches including immunotherapy, probiotic-assisted therapy, anti-enteric secretory drugs, Chinese medicine, and natural compounds. We present the latest advances in the field of rotavirus antivirals and highlights the potential use of Chinese medicine and natural compounds as therapeutic agents. This review provides an important reference for rotavirus prevention and treatment.

## 1 Introduction

**Figure 1:**
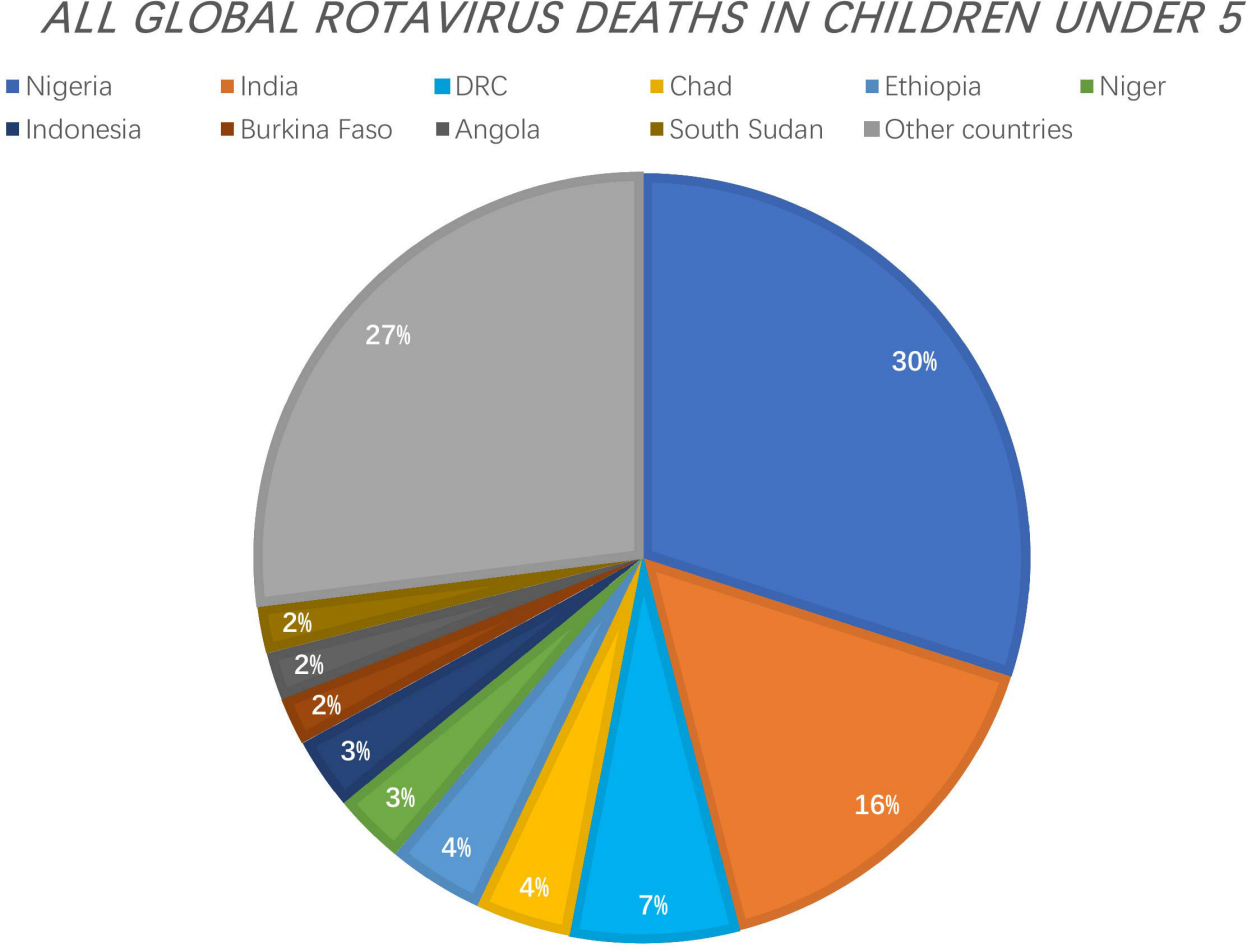
The Countries with the Greatest Number of Rotavirus Deaths as a Proportion of All Global Rotavirus Deaths in Children under 5. 53% of rotavirus-associated deaths worldwide occurred in three countries (Nigeria, India, Democratic Republic of Congo). [https://preventrotavirus.org/rotavirus-disease/global-burden/].

Rotavirus is the major cause of severe diarrhoea in infants and young animals worldwide. It causes 130 million childhood infections and over 200,000 deaths each year and contributes to significant losses to livestock production. In 2006 and 2008, two live oral attenuated rotavirus vaccines - Rotarix^®^ (RV1) and RotaTeq ™ (RV5) - were approved for the prevention of rotavirus infection (1). In 2009, WHO made a recommendation for the global use of rotavirus vaccines, particularly in developing countries with high mortality rates of childhood diarrhoea. Prior to this recommendation, rotavirus gastroenteritis killed approximately 500,000 children under 5 years of age each year. A review of rotavirus deaths among children under 5 years of age from 2000 to 2013 showed that global rotavirus-associated deaths declined from 528,000 in 2000 to 215,000 in 2013. In 2017, 53% of rotavirus-associated deaths worldwide occurred in three countries (Nigeria, India, Democratic Republic of Congo).[https://preventrotavirus.org/rotavirus-disease/global-burden/](**Figure 1**) Two other live oral attenuated vaccines are used for routine vaccination in India: ROTAVAC (Bharat Biologicals) and ROTASIIL (Serum Institute of India), and several other vaccines are in development, including a non-replicating injectable vaccine (2). At present, there are two vaccines in the Chinese market: Rotavirus Vaccine for Live, Oral (Lanzhou Institute of Biological Products), which was marketed in China in 2001, and RotaTeq™ (Merck Sharp & Dohme, USA), which was introduced in 2018 (3). Although the introduction of rotavirus immunization programs has reduced mortality by 60%, vaccine efficacy in developing countries is low (4). Oral and intravenous rehydration remains the mainstay of rotavirus treatment today; however, it does not alleviate the course and severity of the diarrhoea because it is only symptomatic therapy. Therefore, there is an urgent need for the development of specific anti-rotavirus drugs.

## 2 Overview of rotavirus

### 2.1 Epidemiology

Rotavirus is transmitted through faecal-oral transmission and aerosol transmission. The infected hosts are mainly young children and animals, and immunocompromised patients such as those with organ- and stem cell transplantation. The infection sites in the host are the mature villus cells and intestinal endocrine cells (intestinal chromaffin cells) in the upper middle part of the small intestine. Rotavirus infections cause diarrhoea and vomiting symptoms mainly through the destruction of enterocytes (loss of epithelial cell absorption) and activation of the enteric nervous system (ENS) by NSP4 enterotoxin (5). And the viral replication was also found in salivary gland ductal epithelial cells recently (6).

### 2.2 Rotavirus structure

**Figure 2:**
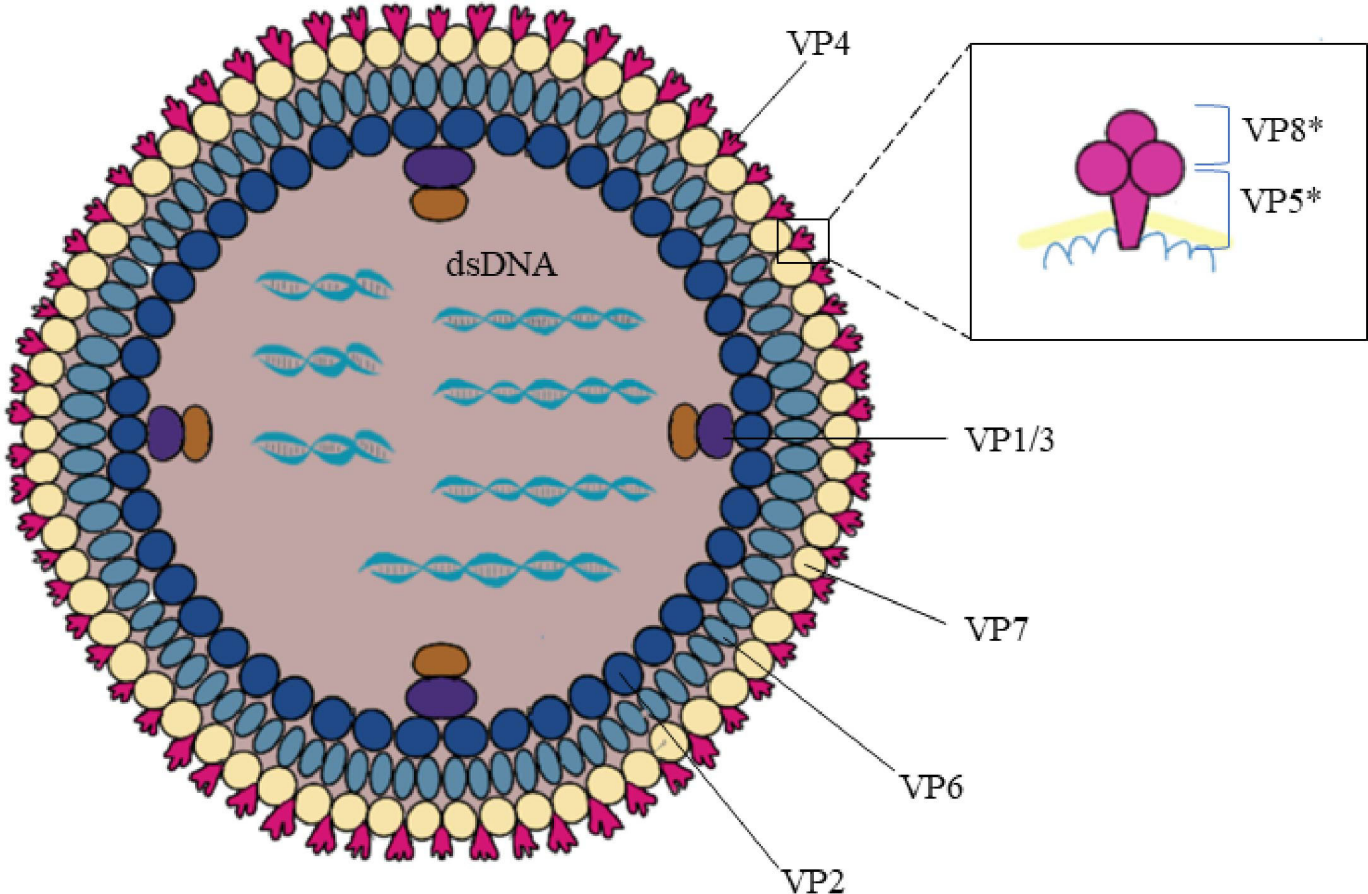
Rotavirus structure. Rotavirus consists of three concentric coat layers surrounded by 11 segmented double-stranded RNA fragments with a genome length of 18,500 bp which encodes 6 structural proteins (VP1-4, VP6-7) and 5-6 non-structural proteins (NSP1-5/6). The capsid structure consists of an inner capsid layer (VP2), a middle capsid layer (VP6), and an outer capsid layer (capsid glycoprotein VP7 and hemagglutinin spike protein VP4). VP4 is cleaved into VP5* and VP8* by the action of trypsin which enhances rotavirus infectivity. The bottom of the VP5* protein is half-buried inside the VP6 protein.

The rotavirus genus belongs to the Reoviridae family and is an envelope-free icosahedral-shaped double-stranded RNA virus with spikes and an average diameter of 100 nm (7). It is named rotavirus because of its wheel-like appearance when viewed by electron microscopy (8). Rotavirus consists of three concentric coat layers surrounded by 11 segmented double-stranded RNA fragments with a genome length of 18,500 bp which encodes 6 structural proteins (VP1-4, VP6-7) and 5-6 non-structural proteins (NSP1-5/6). The capsid structure consists of an inner capsid layer (VP2), a middle capsid layer (VP6), and an outer capsid layer (capsid glycoprotein VP7 and hemagglutinin spike protein VP4) (9). *In vitro* experiments showed that trypsin cleavage of VP4 into VP5* and VP8* is necessary for virus entry into cells. Analysis of the structural domains of VP5* and VP8* reveals some information about the early interaction between the virus and the host cell (**Figure 2**). The viral genome and the viral proteins and functions it encodes are shown in **Table 1**.

**Table 1:** The rotavirus genome and its encoded viral proteins and functions.

Genome fragments	Nucleotides (bp)	Number of copies	Encoded protein	Protein size (kDa)	Function
1	3302	NA	VP1	125	RdRp; binding ssRNA
2	2687	120 (1)	VP2	94	Trigger RdRp to start replication
3	2592	NA	VP3	88	VP1 and VP3 are involved in transcription of the intact intraparticle genome (2); each of the 11 dsRNA fragments is associated with one copy of RNA-dependent RNA polymerase (RdRp), VP1 and the RNA capping enzyme VP3.
4	2362	120 (3)	VP4	88	P-neutralizing antigen (4); receptor-binding and cell penetration (3)
	NA	NA	VP5*	60 (5)	VP5* contains a hydrophobic structural domain that promotes rotavirus penetration into cells (2). The VP5* subunit exposes the recognition sequence of α2β1 integrin, which is associated with post attachment by binding to heat shock protein 70 (HSP70) and integrin α2β1 (6).
	NA	NA	VP8*	28	VP8* contains a hemagglutination- or sialic acid binding domain which is necessary for rotavirus subgroups that require sialic acid for infection (2, 3).
5	1581	NA	NSP1	58	Mediates nuclear factor kappa-light-chain-enhancer of activated B cells (NF-κB) activation to inhibit the type I interferon (IFN) response; mediates degradation of interferon regulatory factors (7).
6	1356	780 (1)	VP6	44	Contains serotype-specific antigens which is the basis for strain typing (8).
7	1062	780	VP7	37	Receptor-binding; G-type neutralizing antigen (4); calcium-binding protein; post-attachment interacts with cellular receptors (3, 8).
8	1059	NA	NSP2	36	Involved in virion formation
9	1074	NA	NSP3	34	Promotes RV mRNA transcripts
10	751	NA	NSP4	20	Enterotoxins, the main cause of enterotoxicity
11	666	NA	NSP5	21	Involved in virion formation
	NA	NA	NSP6	12	RVC do not encode NSP6

*In vitro*, VP4 is cleaved into VP5* and VP8* by the activation of trypsin.

### 2.3 Rotavirus serotyping

Rotavirus is classified into 7 serotypes (A-G) according to the difference in VP6 serotypes, with A, B, and C infectious to humans. Rotavirus outer capsid proteins VP4 and VP7 play an important role in virus adsorption and entry into host cells and have neutralizing antigens; The VP7 (G) and VP4 (P) antigens can be freely combined, hence, there is a dual classification scheme: VP7 (G1-G36), VP4 P[1]-P[51], at least 51 species) (3, 10, 11).

## 3 Research progress of anti-rotavirus drugs

There are no FDA-approved drugs for rotavirus gastroenteritis. Most deaths are associated with excessive water and electrolyte loss due to vomiting and diarrhoea; therefore, oral intravenous rehydration is the most basic treatment strategy. Probiotics including Lactobacillus rhamnosus GG(LGG) are recommended by several guidelines around the world (12). In addition to anti-RVA secretory IgA (13, 14), breast milk is rich in oligosaccharides and proteins that inhibit rotavirus infection of the host and modulate host immune function, therefore, breastfeeding is widely recommended (15). A article published in 1982 reported that rotavirus antibodies were found in the fresh milk but not after pasteurization (16). Racecadotril is an anti-secretory drug used as an adjunct drug for acute gastroenteritis treatment yet it is not promoted; some studies support its therapeutic efficacy, while other paediatricians believe that Racecadotril with oral rehydration is ineffective (17). The inclusion of rotavirus vaccine in immunization programs has decreased reported rotavirus infections worldwide by 60%. However, deaths are still as high as 200,000 and 90% of fatal rotavirus infections occur in low-income countries where vaccine coverage is low and access to care for children is limited. This calls for the urgent development of specific anti-rotavirus drugs.

### 3.1 Rotavirus replication cycle

**Figure 3:**
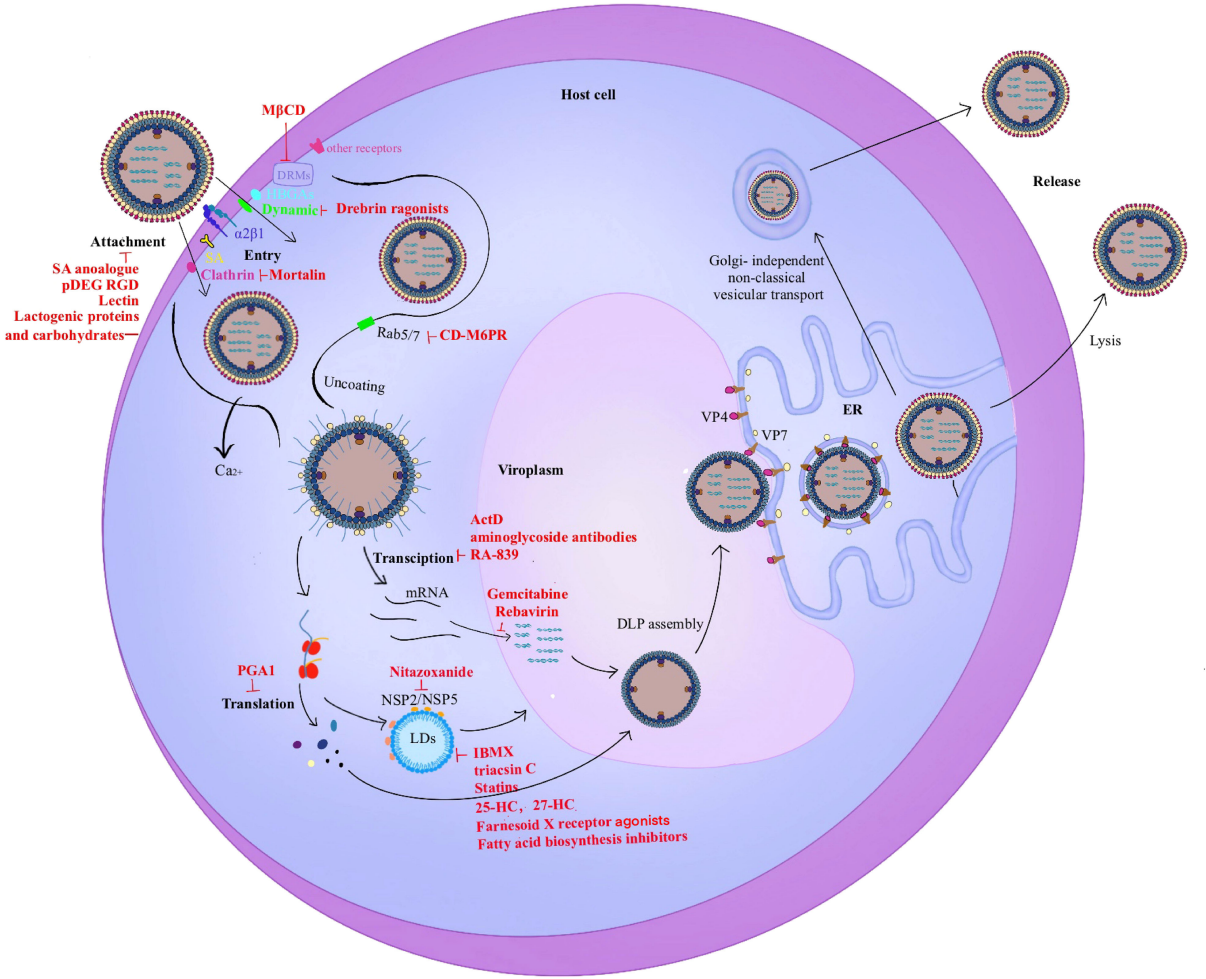
Rotavirus life cycle. The entry stage of rotavirus replication is complex. 1). The first step in infection is the recognition of rotavirus and host surface molecules, including sialic acid, α2β1, and histo-blood group antigens (HBGAs). Rotavirus invades cells through two different endocytosis pathways (clathrin-dependent or dynamin-dependent, depending on the strain), and a mode of direct penetration. VP5* contains a hydrophobic structural domain that promotes rotavirus penetration into cells. 2). Triple-layered particles (TLPs) entering the cytoplasm enter the same endosome and migrate from early endosomes (EEs) to mature endosomes (MEs) and late endosomes (LEs) with Rab5/7 transformation. MEs exist in Rab5 and Rab7. As the Ca^2+^ level decreases, the viral outer capsid disintegrates and double-layered particles (DLPs) are released into the cytoplasm which triggers viral translation and replication. 3). The viroplasm is the main site of rotavirus genome replication and packaging. The interaction of NSP2/NSP5 with lipid droplets (LDs) is the first step in the formation of viral plasmids. 4). The virus precursor DLPs completes the outer capsid assembly in the endoplasmic reticulum (ER). 5). Mature viral particles are released by cell lysis in polarized cells such as monkey embryonic kidney cells MA-104. In contrast, mature viral particles are released by budding in non-polarized cells such as human intestinal cells Caco-2 which does not cleave host cells.

The rotavirus replication cycle is the main development direction of antiviral drugs. The related compounds targeting different stages of the life cycle are shown in **Figure 3**.

#### 3.1.1 Drugs inhibiting viral adherent cells

Rotavirus attachment to host cells is dependent on trypsin-like enzyme cleavage of viral outer capsid proteins VP4 and VP7 in the intestine to become infectious. This process cleaves VP4 into the C-terminal VP5* domain and the N-terminal VP8* domain, which contains the hemagglutination- or sialic acid binding domain necessary for the subgroup of rotaviruses that require sialic acid for infection (9, 18). Sialic acid is usually in the form of oligosaccharides, glycolipids, or glycoproteins, and binds directly to rotavirus to inhibit viral adhesion; however, this inhibition appears to be strain-dependent (19). Mucin is a negatively charged glycoprotein that is a major component of the mucus layer of the gastrointestinal tract (20). Supplementation with leucine (21) or LGG (22) promotes mucin production. The sialic acid content of mucin is proportional to its antiviral activity (23). The antiviral activity of sialic acid glycoprotein is demonstrated *in vitro* and *in vivo* (24). Many studies observed that salivary acid mimics inhibited rotavirus replication *in vitro* and *in vivo* (25–27). Liakatos A et al. (25) also showed that lactose-based salivary acid mimics were not inhibitors of rhesus rotavirus strains; however, they exhibited moderate inhibition of human rotavirus Wa strain. The neoglycolipid receptor mimic is a synthetic neoglycolipid containing sialic acid lactose. Lactose sialate is the carbohydrate fraction of GM(3) and GM(3) ganglioside receptors are required for host cell recognition by sialic acid-dependent rotavirus. However, GM(3) ganglioside production as an oral therapeutic agent in pigs is costly. The new glycolipid receptor mimic reduces rotavirus infection of host cells by over 90% using concentrations comparable to GM(3) (28). Integrin α2β1 also plays a key role in the early attachment step of infection, and an α2β1 integrin-binding motif is located in the N-terminal region of donkey lactamucin (29). A peptide containing 20 amino acids of the DGE and RGD motifs (pDGE RGD) exhibited significant antiviral activity. Thus, the authors propose developing anti-rotavirus peptides that block viral receptor binding. A similar effect was observed with ricin lectin, a galactose-specific lectin that competitively binds to monkey rotavirus SA11 and inhibits infection at concentrations as low as 10^-9^ M (30). A neutralizing effect of bovine lectins on rotavirus infectivity was also observed (31).

Oligosaccharides are abundant in breast milk and dairy products (32). Their structure is similar to that of viral receptors and can trap viruses and prevent viral adhesion to host cells (33, 34). Human rotavirus infectivity of strains Wa and DS1 in MA104 cells is inhibited by milk oligosaccharides. Laucirica DR et al. showed that the reduction in infectivity by oligosaccharides was mainly achieved through effects on the virus rather than that on the cells. The authors suggest that breastfeeding reduces rotavirus infection and propose adding specific oligosaccharides to infant formulas (15). Recently, breast milk oligosaccharides (2’-FL, scGOS/lcFOS) modulate the gut microbiota in neonatal rats and regulate cellular Toll-like receptor (TLR) gene expression, suggesting that breast milk oligosaccharides prevent rotavirus infection (35). Breast milk oligosaccharides (2’-FL, LNnT, 6’-SL, and 3’-SL) modulate the gut microbiota and stimulate the immune response to rotavirus in piglets (36). In addition, the anti-rotavirus activity of some major breast milk proteins (milk fat globule membrane, whey protein concentrate, and lactoferrin) appears to modulate the immune response in young rats (37, 38). Lactoferrin is an iron-binding protein enriched in mammalian milk and has several physiological functions (antibacterial, anti-inflammatory, immunomodulatory). It has antiviral activity against DNA- and RNA viruses including rotavirus, respiratory syncytial virus, herpesvirus, and human immunodeficiency virus (HIV) (39). The anti-rotavirus activity of lactoferrin occurs during the pre-attachment and entry phase of the virus (40, 41), and prevents the virus from entering the host cell by blocking cellular receptors or binding to viral particles (42). It is agreed that lactogenic proteins and carbohydrates are the main active components against pathogens (20, 43, 44). Subsequently, studies have shown that children who drink low-fat milk are 5 times more likely to visit a doctor for acute diarrheal disease compared to those who drink whole milk. Buttermilk MFGM containing polar lipid enrichment exhibits a greater rate of rotavirus inhibition than that of whey MFGM (45). Therefore, lipids such as sphingolipids and triglycerides may have antiviral- and antibacterial effects and prevent foodborne gastroenteritis.

The charged properties of polymers also seem to influence viral infectivity. Negatively charged mucins, heparin, acetyl heparin sulphate, alpha-1-acid glycoprotein, dextran sulphate, and glyoxylate (stevia extract) inhibit viral replication (46). These negatively charged polymers appear to interfere with the binding of VP7 and cellular receptors through spatial site-blocking. However, positively charged polymers (fisetin, fisetin sulphate, DEAE-dextran, and histones) enhance viral infection (47).

#### 3.1.2 Drugs inhibiting virus entry into cells

After rotavirus attachment to the host cell surface, TLPs can enter the cell by different endocytic pathways or by directly penetrating the cell membrane. Although rotavirus is a non-enveloped, large hydrophilic viral particle, the virus appears to rely on the presence of a hydrophobic structural domain of VP5* after VP4 cleavage for direct cell entry after viral attachment to the cell surface (48–50). This cleavage process does not affect cell binding.

In general, viruses can undertake endocytic pathways including clathrin-mediated endocytosis, caveolae-mediated endocytosis, phagocytosis, and dynamin-dependent endocytosis (51). Using two rotavirus strains infecting monkey, RRV and SV40, Claudia Sa´nchez-San Martí et al. described that the important role of Dynamin in the entry of these two rotavirus strains in MA104 cells. However, later studies have shown that the endocytic pathway of rotaviruses appears to be strain dependent. VP4 appears to determine the endocytic pathway of rotavirus entry into MA104 cells, and the infectivity in MA104 cells was determined using a set of UK × RRV recombinant viruses with different combinations of viral structural proteins. Human rotavirus strain Wa, porcine strain TFR-41, and bovine strain UK appear to enter cells *via* clathrin-mediated endocytosis, while strain RRV is sensitive to cellular neuraminidase (NA) processing using integrins α2β1 and αvβ3, and heat shock cognate protein 70 (hsc70) as receptors, and is dependent on kinesin and a non-lattice protein-mediated pathway in the presence of cell surface cholesterol (52). The same requirement for hsc70, dynamin, and cholesterol was confirmed in independent experiments, although endocytoses in RRV is different from that of other strains (53).

Cholesterol in the plasma membrane is necessary for rotavirus infection. Lipid rafts are micro-regions within the plasma membrane bilayer that are rich in cholesterol and have higher lipoprotein ratios. The infectivity of PRV and RRV is significantly reduced in cells treated with methyl-β-cyclodextrin (MβCD) (54, 55). The lipid raft structure has no significant effect on virus attachment and mainly controls the entry phase of the virus. This suggests that reduction of the cholesterol content in the plasma membrane may help fight against rotavirus infection. Lipid raft structures are also known as detergent-resistant membrane domains (DRMs). Ganglioside GM1, integrin subunits α2 and β3, and hsc70 are rich in DRMs. The virus is excluded from the DRM if cells are treated with MβCD (55), suggesting that cholesterol-rich membrane lipid microdomains provide a platform for efficient attachment of rotaviruses and cellular receptors. And not all rotaviruses require integrins, but all tested rotaviruses require hsc70.

Drebrin is a cytoskeletal protein that inhibits dynamin mediated endocytosis, including the early entry steps of rotavirus, vesicular stomatitis virus (VSV), human adenovirus 5 (HAdV5), and SV40. It restricts the entry of multiple viral pathogens, including rotavirus into host cells (56). Since genetic deletion- or chemical inhibition of Drebrin increases rotavirus infection *in vitro* and increases diarrhoea incidence and viral shedding *in vivo*. The authors propose that Drebrin agonists should be developed for antiviral drugs. Clathrin is a trimer of heavy chain polypeptides (CLTC) and each heavy chain subunit binds a light chain subunit. Clathrin-mediated endocytosis is a well-characterized endocytic pathway and a major route of entry for rotaviruses other than RRV into cells. Mortalin is a member of the heat shock protein HSP70 family, and its carboxyl terminus interacts with the CLTC of clathrin (57). Mortalin induces CLTC degradation *via* the proteasome pathway, thereby inhibiting the clathrin-mediated endocytic pathway into host cells.

The receptor tyrosine kinase inhibitors (RTKIs) AG879 and tyrphostin A9 (A9) exhibit *in vitro* inhibitory activity against various RNA- and DNA viruses, including Sendai virus (Paramyxoviridae), herpes simplex virus (Herpesviridae), mouse hepatitis virus (Coronaviridae), and rhesus rotavirus (Reoviridae) (58). The receptor tyrosine kinase is an enzyme and a receptor capable of binding to a ligand and phosphorylating the tyrosine residues of the target protein. Rotavirus NSP5 is thought to have kinase activity and the replication process of rotavirus causes a series of phosphorylation changes. Genistein antiviral activity against monkey rotavirus strain SA11 (an bovine RF), bovine UK, Rhesus RV, nar3, porcine rotavirus strain YM, and human WA showed that rotavirus infectivity is strain-dependent (59). RTKIs reduce the affinity of integrins to bind rotavirus by blocking the “inside-out” signalling of integrins through the inhibition of intracellular receptor tyrosinase (60), which suggested that the inhibition of rotavirus replication by genistein may be achieved by upregulating AQP4 expression through the cAMP/PKA/CREB signalling pathway.

Protein disulphide isomerase (PDI) is an oxidoreductase present in mammalian cells that acts as a reducing agent in the cell membrane; it reduces the cell membrane-binding protein disulphide bond. DTNB [5,5’-dithiobis-(2-nitrobenzoic acid)] and bacteriocin inhibit the redox activity of PDI. Thiol/disulphide exchange is involved in the rotavirus entry process and redox reactions affect rotavirus infectivity. DTNB, bacitracin, and anti-PDI antibodies reduce the infectivity of rotavirus in MA104 cells (61). Comparison of anti-rotavirus drugs ibuprofen, NAC, and pioglitazone which all interfere with the NF-kB pathway showed that NAC was the most promising drug for the treatment of rotavirus infection in children, and the expression level of hsc70 and PDI in cells of the drug-treated group returned to the level of the uninfected virus group (62). Clinical data show good therapeutic efficacy of N-acetylcysteine for rotavirus infection (63). The Nrf2/ARE pathway is sensitive to cellular redox stress, and RA-839 is a recently identified agonist of the Nrf2/ARE pathway. RA-839 inhibits viral RNA and protein translation, while the anti-rotavirus activity of RA-839 is supported by two classical pharmacological activators of the Nrf2/ARE pathway (2-cyano-3, 12-dioxooleana-1, 9(11)-dien-28-oic acid methyl ester (CDDO-Me) and hemin) (64).

Rotaviruses use different endocytic pathways to enter cells. In most cases, these viruses enter EEs, while some viruses enter the cytoplasm from EEs to initiate zygotic replication. The pathways of intracellular transit are different for different strains, with hominin RRV and SA11 reaching the cytoplasm from MEs, while bovine UK, porcine TFR-41, and human Wa travel from MEs to LEs to the cytoplasm (59). Rab proteins are molecular switches in cellular vesicle transport; EEs and LEs are enriched in GTPase Rab5 and GTPase Rab7, respectively. Most rotavirus strains must be transported to LEs, and Rab5 inhibition blocks RRV infectivity, while Rab7 inhibition blocks BRV- UK infectivity. Most virulent strains need to enter the cytoplasm *via* LEs to acquire infectivity and require CD-M6PR (cation-dependent mannose-6-phosphate receptor) mediated histone protease activity; therefore, this receptor may be used as a drug target (65).

Prostaglandin E_2_ is an abundant eicosanoid produced from arachidonic acid during rotavirus infection and the site of production is the lipid droplet. Epoxygenase is necessary for prostaglandin (PG) biosynthesis. Indomethacin has an anti-rotavirus effect against human rotavirus Wa or simian rotavirus SA-11 in Caco-2 cells during the post-attachment phase of the viral infection cycle to reduce viral protein synthesis and does not affect viral RNA synthesis (66). Prostaglandin A1 (PGA1) may prevent SA-11 rotavirus maturation in MA-104 cells by inhibiting NSP4 glycosylation or VP4/VP7 synthesis (67). Prostaglandin E_2_ inhibitors indomethacin, CAY10502, celecoxib, and SC-560 affect SA11 viral internalization *in vitro* in early infection and VP6 RNA synthesis in the mid-stage in MA-104 cells (68). Furthermore, prostaglandin E_2_ replacement enhances rotavirus infection.

Multiple group A rotavirus strains including SA11 (genotype P[2]), Wa (genotype P[8]), DS-1 (genotype P[4]), and Azuk-1 (genotype P[29])-over-expressing type II transmembrane serine protease (TTSP) cells are not dependent on trypsin activation and TMPRSS2 and TMPRSS11D are potential targets (69). However, whether TTSP is involved in group A rotavirus infection in the presence of trypsin remains to be explored.

Gangliosides are glycosphingolipids composed of ceramide and contain one or more sialic acid residues. Downregulation of ganglioside expression reduces the infectivity of the four rotavirus strains tested: human Wa, simian RRV, porcine TFR-41, and bovine UK. Gangliosides did not affect early viral binding and post-entry replication (70) which contradicts previous findings (28). In contrast, a new glycolipid receptor mimic inhibits 90% of porcine rotavirus OSU strain and MA-104 cell binding. The reason may be that the virus relies on other sialic acid-containing cellular molecules, histo-blood group antigens (HBGAs), or other glycoproteins to bind to cells (71, 72). Further studies are required to determine whether gangliosides prevent viral recognition of cells to reduce viral infectivity.

#### 3.1.3 Inhibition of genome replication

It is widely believed that trypsin-like proteases promote rotavirus replication *in vivo* and *in vitro*, and protease hydrolysis causes PDCoV (73), TuMV (74), influenza A and B viruses (75), reovirus, and rotavirus (76). The small intestine is the main site of rotavirus replication where there are high levels of protease activity. Protease inhibitor blocks virus replication when added at different times after rotavirus inoculation, inhibits VP4 activation, and prevents intercellular transmission of the virus (77).

A decrease in calcium ion concentration enhances viral infection. The removal of calcium ions from the endosome disintegrates the outer layer of TLPs and releases DLPs into the cytoplasm to activate VP1 and VP3 and trigger viral ssRNA replication and transcription (7, 78). Chelators such as EDTA and EGTA remove Ca^2+^ from viral particles (79); EDTA treatment disrupts the rotavirus outer capsid and hinders infectivity.

#### 3.1.4 Inhibition of protein translation

Hsp90 inhibitors affect the formation of functionally active mature NSP3 (dimer) by blocking the direct interaction between Hsp90 and NSP3, which causes nuclear translocation of poly(A)-binding proteins and reduces viral protein translation (80).

Actinomycin D (Act D) is an antibiotic embedded in DNA that inhibits DNA-dependent transcription. It may bind to viral RNA and prevent newly synthesized mRNA from being translated; thereby inhibiting SA-11 replication in MA-104 cells (81). Neomycin B inhibits rotavirus proliferation by affecting the transcription- and replication of the viral genome. Other aminoglycoside antibiotics including levamycin, palomycin, and tobramycin have similar antiviral effects (82). Although numerous antibiotics have significant antiviral effects against rotavirus, their use should be limited due to side effects such as accelerated virus evolution, disruption of normal human flora, and damage to kidney function.

#### 3.1.5 Drugs inhibiting intracellular viral replication

Rotaviruses use energy and raw materials from host cells and rely on their own VP1 (RdRp) to complete viral genome replication. Many broad-spectrum antiviral drugs target RdRp to inhibit viral replication by interfering with nucleotide synthesis, including nucleotide analogues (gemcitabine) (83) and nucleoside analogues (ribavirin) (84–88).

#### 3.1.6 Drugs inhibiting viroplasm formation

The viroplasm is the main site of rotavirus genome replication and packaging. Its formation is associated with LDs and viral proteins, especially NSP2 and NSP5. After translation of viral proteins, LDs interact with NSP2 as the first step in viroplasm formation (89). Therefore, drugs that directly target LDs or viroplasm formation are a viable antiviral strategy.

Nitazoxanide is a nitrothiazole derivative of salicylamide (90) that was initially developed as an antiparasitic drug. It has broad-spectrum antiviral activity (91), and is clinically effective in the treatment of rotavirus-induced diarrhoea (92, 93). The combination of nitazoxanide and probiotics significantly reduces the duration of diarrhoea in children infected with rotavirus (94). Thiazolides interferes with viral NSP2/NSP5 interactions to affect viroplasm formation and size alteration, thereby reducing dsRNA formation (89).

Lipid droplets are organelles involved in lipid metabolism and are responsible for the storage of triacylglycerols, cholesterol, and cholesteryl esters. The relationship between LDs and viroplasm is prompted by the insertion of phosphoproteins in a cyclic form on their surfaces. β-adrenergic agonist isoproterenol and the phosphodiesterase inhibitor isobutylmethylxanthine (IBMX) promotes the degradation of LDs and catecholamines, with the hormones involved in this process playing an important role. Triacsin C is a specific inhibitor of long chain acyl coenzyme A synthetases that blocks LD formation. The above compounds prevent rotavirus infection by reducing the amount of viroplasm to inhibit dsRNA replication and mature viral particle production (95). In recent years, the importance of lipid metabolism for virus-host interactions was recognized for hepatitis C virus (HCV), dengue virus, GB virus B, flavivirus, bunyavirus, intracellular parasites Chlamydia, and SARS-CoV-2 (96, 97).

Bile acids and synthetic farnesoid X receptors (FXR) play important roles in lipid metabolism. Bile acids and synthetic FXR agonists reduce rotavirus infection by downregulating lipid synthesis *in vitro* and *in vivo* (98). In addition, statins are widely known as lipid-lowering agents that reduce total cholesterol levels by competitively inhibiting the endogenous cholesterol synthesis rate-limiting enzyme HMG-CoA reductase and blocking the intracellular hydroxymevalonate metabolic pathway. Statins may also have beneficial effects for the treatment of rotavirus infections. Rotavirus replication is significantly inhibited after statin treatment (99) or HMGCR knockdown (100). Notably, viral titres in the drug-treated group of cells decrease by 2 log values while viral mRNA remains relatively unchanged, suggesting that statins affect viral assembly. Similarly, oxysterols are cholesterol oxidation derivatives involved in cholesterol biosynthesis. 25-hydroxycholesterol (25-HC) and 27-hydroxycholesterol (27-HC) have significant antiviral activity against three non-enveloped viruses, including (human papillomavirus-16 (HPV-16), human rotavirus (HRoV), and human rhinovirus (HRhV) (101). Meanwhile, hydroxysterols from breast milk may have antiviral properties (102). Fatty acid biosynthesis is important for lipid homeostasis, and neutral fats stored in LDs are also synthesized by this pathway. Treatment with fatty acid biosynthesis inhibitors results in a 3.2-fold decrease in rotavirus titre and a 1.2-fold decrease in viral RNA level, suggesting a role for LDs in rotavirus assembly or release (103).

#### 3.1.7 Inhibition of virus maturation

The endoplasmic reticulum is the site of viral maturation where viral precursor DLPs formed by virion assembly outgrow across the endoplasmic reticulum, briefly acquire an envelope, acquire VP4 (60 trimers) and VP7 (260 trimers), then remove the envelope to complete the assembly of mature infectious TLPs. Mature viral particles are released by cell lysis in polarized cells (such as MA-104). Meanwhile, mature viral particles are released by an unconventional vesicular transport mechanism in non-polarized cells (such as Caco-2): direct transport from the endoplasmic reticulum to the cytoplasmic membrane without passing through the Golgi complex, in a non-cleaved host cell budding manner (104).

### 3.2 Immunotherapy

**Figure 4:**
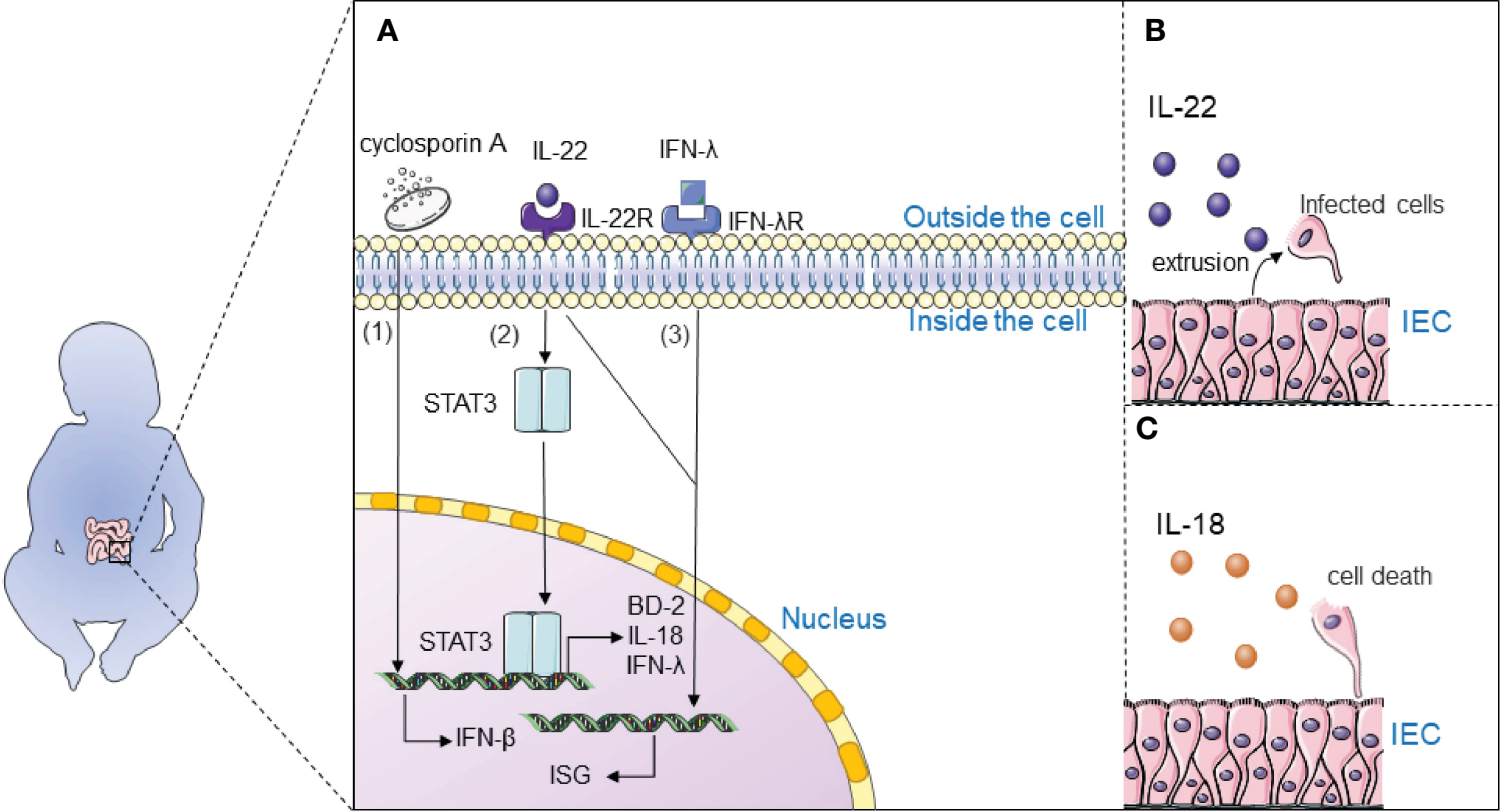
Immunotherapy inhibits rotavirus infection. **(A)** 1) Cyclosporine A may inhibit rotavirus infection by regulating the expression of IFN-β to activate the intracellular IFN1-based immune response; 2) Interleukin-22 (IL-22) inhibits rotavirus infection by activating the STAT3 signaling pathway and upregulates the expression of antimicrobial genes in the gut, including antimicrobial peptide β-defensin (BD-2), cytokine IL-18, and interferon-λ (IFN-λ); 3) Interferon-λ and IL-22 synergistically induce interferon-stimulated genes (ISG) and control rotavirus infection; **(B)** IL-22 extrudes cells from tissues by stimulating the proliferation of infected intestinal epithelial cells (IECs) and their migration toward the tips of the villi; **(C)** IL-18 promotes death of infected IECs to directly interrupt the viral replication cycle.

Immunotherapy may complete the long-term control of the virus without specific antiviral therapy by relying on the patient’s own immunity. Therefore, drug development to activate the cellular immune response, modulate the inflammatory response, and further restore the adaptive immunity of the body is a therapeutic strategy (**Figure 4**).

It is widely believed that interleukin-22 (IL-22) inhibits rotavirus infection by activating the STAT3 signaling pathway and upregulates the expression of antimicrobial genes in the gut, including antimicrobial peptide β-defensin (BD-2), cytokine IL-18, and interferon- λ (IFN-λ) (105, 106). TLR5-mediated IL-22- and NLRC4-mediated IL-18 cytokines induce the expression of each other and inhibit the replication of rotavirus. IL-22 extrudes cells from tissues by stimulating the proliferation of infected intestinal epithelial cells (IECs) and their migration toward the tips of the villi. IL-18 promotes death of infected IECs to directly interrupt the viral replication cycle. Combination therapy with IL-18 and IL-22 may be a treatment for rotavirus infection since IL-22 and IL-18 can induce expression of one another. Interestingly, IL-22 and IFN-λ have a close relationship: Il22ra1 encoding the α chain IL-22 receptor, and Ifnlr1 encoding the IFN-λR1 chain, are closely located on human and mouse chromosomes; IL-22Rα and IFN-λR1 are associated with the IL-10Rβ chain (also known as IL-10R2) to form functional heterodimeric receptor complexes. Interferon-λ and IL-22 synergistically induce interferon-stimulated genes (ISG) and control rotavirus infection (107). It is generally accepted that IFN plays an important role in antiviral defence; Type 3 III plays an important role in rotavirus infection and induces type I IFN to some extent (108). Type I IFN induces the expression of ISGs through activation of the JAK/STAT signaling pathway, which induces entry into the antiviral state. Natural human IFN-α successfully treats rotavirus infection in neonates and piglets (109).

Organ transplant patients and other immunocompromised individuals are more susceptible to rotavirus infection, and the choice of immunosuppressant is critical for their treatment. Cyclosporine is a commonly used immunosuppressive agent in clinical practice. It is proposed that cyclosporine A may inhibit rotavirus infection by regulating the expression of cytokines (especially IFN-β) to activate the intracellular IFN1-based immune response (110, 111). Mycophenolic acid (MPA) has anti-rotavirus activity as an immunosuppressant, and it inhibits IMPDH (inosine-5’-phosphate dehydrogenase) activity which can lead to guanylate depletion and inhibition of rotavirus replication (112). However, rotavirus resistance to MPA is a barrier for drug application. mTOR inhibitor rapamycin inhibits PI3K-Akt-mTOR signaling and activates autophagy which facilitates anti-rotavirus effects (113). A similar conclusion was reached in another study which identified a virus-like small RNA (RV-vsRNA1755) that inhibits PI3K-Akt-mTOR signaling and activates autophagy (114).

Evidence has shown that immunoglobulins derived from bovine colostrum, egg yolk of immunized hens, or human pooled plasma may be promising passive immunotherapy for children, but not neonates. Llama-derived heavy chain antibody fragments (VHHs), designated as ARP1 and ARP3 (ARP: anti-rotavirus protein) was shown to significantly reduce stool output in severe cases of infantile rotaviral diarrhea (115). SusanaLópez et al. found a hybridoma secreting a monoclonal antibody (MAb) (2D9) can specifically block the infectivity of wild-type RRV and its sialic acid-independent variant nar3 (116).

### 3.3 Intestinal function

The infection of rotavirus triggers watery diarrhea. The mechanisms for the potential drugs targeting intestine have been intensively studied (**Table 2**).

**Table 2:** Drugs increasing intestinal barrier function.

Drugs	Class(es) of inhibitor(s)	Antiviral mechanism
LGG,Bifidobacterium, Lactobacillus, Lactobacillus acidophilus and Lactobacillus reuteri)	probiotics	Regulates the immune response of the host;properly blocking rotavirus infection by interacting directly with the virus.
Racecadotril	ENS inhibitors	Inhibits enkephalinase activity and preventing intestinal secretion.
Plumbagin, Trans-δ-glucoside and iOWH032	Cl^-^ channel inhibitors	Inhibits Cl^-^ channel associated secretory diarrhea
Ondansetron	5-HT receptor antagonist	Repairing ENS damage caused by RV
Er xie ting granules, lunxieting paste and Qiwei Baizhu powder	Chinese medicine	Alleviates pathological movement of the small intestine
P2Y1 inhibitors, Tunicamycin,brefeldin A,CaM and BAPTA-AM	Ca^2+^ channel inhibitors	Targets plasma membrane Ca^2+^ permeability
transmontane saponite	Antidiarrheal	Blocks the interaction with enterocytes by trapping RV
lentinan	oligosaccharide	Improves intestinal microbiota and reducing intestinal cell apoptosis
transforming growth factor-α	Polypeptide growth factor	Repairs damaged intestinal mucosa

#### 3.3.1 Probiotics

Probiotics are active microorganisms that exert beneficial effects by modulating the host’s intestinal flora or interacting with the host’s immune system. Growing evidence supports the idea that probiotics are effective in the prevention and treatment of rotavirus infections. The use of *L. rhamnosus* GG (LGG) and Saccharomyces boulardii as supplemental therapy with oral rehydration solutions is recommended by several guidelines around the world and the anti-rotavirus activity of LGG is widely studied (12, 117–119). The anti-rotavirus activity of LGG was first discovered in 1995 (120). LGG rapidly alleviates secretory diarrhoea within a few hours by inhibiting chloride secretion. Long-term LGG supplementation is thought to have a postbiotic effect through regulating intestinal flora, protecting enterocytes, and relieving osmotic diarrhoea (121). Lactobacillus acidophilus, Lactobacillus reuteri, and Bifidobacterium modulate the immune response to rotavirus infection (122–125) and appear to block rotavirus infection by directly interacting with the virus (126).

#### 3.3.2 Suppression of intestinal secretory diarrhoea

Racecadotril inhibits intestinal secretion through selective- and reversible inhibition of enkephalinase activity. Many clinical trials have successfully used Racecadotril to treat acute gastroenteritis in children, including rotavirus gastroenteritis (127–129). It is widely believed that secretory diarrhoea is due to activation of the enteric nervous system (ENS), and rotavirus NSP4 is an enterotoxin that stimulates an increase in intracellular calcium concentration which triggers the release of amines or peptides in the gut, and further activates the enteric secretory nerves. Therefore, inhibition of the ENS may be a target for drug development. Four drugs that inhibit ENS function (tetrodotoxin, lidocaine, hexamethonium, and mecamylamine) all significantly inhibit rotavirus-induced enteric secretion (5).

Chloride (Cl^-1^) channel inhibitors may be an antisecretory and antimotor drug for the widespread treatment of diarrhoea. Rotavirus enterotoxin NSP4 activates transmembrane protein 16A (TMEM16A) and inhibits Na^+^ absorption to induce diarrhoea. TMEM16A inhibitors such as leukodendrin block TMEM16A-mediated calcium activation of Cl^-^ currents. Plumbagin inhibits secretory diarrhoea in neonatal mice infected with rotavirus by 50% (130). Similarly, trans-δ-glucoside prevents secretory diarrhoea in mice by inhibiting TMEM16A-mediated Cl^-^ currents (131, 132), while iOWH032 is a synthetic Cl^-^ channel inhibitor used to treat secretory diarrhoea (133).

Serotonin secretion is Ca^2+^ dependent, and calcium ions, 5-hydroxytryptamine, and gastrointestinal hormone ions may interact to influence the ENS (134). 5-hydroxytryptamine (5-HT) and vasoactive intestinal peptide (VIP) are involved in rotavirus-induced gastroenteritis (135). Ondansetron is a 5-HT receptor antagonist, and the FDA states that it is safe for use in children as young as 1 month old (136). Animal studies and clinical trials have confirmed its efficacy in rotavirus gastroenteritis treatment (137, 138). In addition, clinical observations of er xie ting granules (139) and lunxieting paste (140) showed good results for rotavirus enteritis treatment in children and infants and high compliance in children.

Rotavirus infection causes intercellular signaling which manifests as intercellular calcium waves (ICW) (140). This calcium signal is caused by the release of ADP which activates the purinergic receptor P2Y1 in neighbouring cells and induces the secretion of chloride and 5-hydroxytryptamine, leading to diarrhoea and vomiting. The authors blocked intercellular calcium waves by P2Y1 antagonists or by knocking out the P2Y1 receptor gene (141). Secretory diarrhoea caused by rotavirus is induced by enterotoxin NSP4. Enterocytes are stimulated by NSP4 early in the infection to increase chloride ion and water influx into the intestinal lumen. This causes an increase in intracellular Ca^2+^; therefore, targeting plasma membrane Ca^2+^ permeability may be a target for drug development. Viral protein glycosylation and calcium channel formation is blocked using clathrin and brefeldin A to prevent the formation of infectious viral particles (142). Calmodulin (CaM) interacts with viral protein VP6 in the presence of Ca^2+^ to promote viral replication, and CaM inhibitor (W-7) and Ca^2+^ chelator (BAPTA-AM) reduces the formation of infectious viral particles.

#### 3.3.3 Increasing intestinal barrier function

Rotavirus NSP4 causes damage to the intestinal barrier; therefore, drugs that protect intestinal cells from viral invasion should be considered. The anti-diarrheal effect of transmontane saponite is demonstrated (143). Recently, a study reported that diosmectite blocks the interaction with enterocytes by trapping SARS-CoV-2, thus preventing NF-κB activation (144). The relief of diarrhoea in rotavirus infected piglets by lentinan administration may be the result of improved intestinal microbiota and attenuation of apoptosis (145). Animal tests of Qiwei Baizhu powder confirmed that the drug reduces pathological damage such as small intestinal mucosa and villi damage and improves small intestinal absorption (146). Oral administration of transforming growth factor alpha promotes the recovery of mucosal damage induced by rotavirus enteritis in pigs (147).

### 3.4 Chinese medicine and natural compounds

Natural compounds are natural treasures, and many natural products with pharmaceutical activity (antiviral, anti-inflammatory and antibacterial) were developed and applied (**Table 3**). Shenling Baizhu powder (SBP) mainly composed of Panax ginseng C.A.Mey (Ren Shen), Wolfiporia cocos(Fu Ling), Atractylodes macrocephala Koidz(Bai Zhu), is a classical prescription medicine in Chinese medicine for the treatment of digestive tract diseases. The antiviral mechanism of SBP was investigated based on the effective curative effect of SBP on rotavirus enteritis in clinical practice (148). The protein-protein interaction (PPI) network analysis suggests that the TLR4/MyD88/NF-κB signalling pathway may be activated and SBP treatment restores the upregulation of inflammatory factors and downregulation of IFN-β due to rotavirus infection. GeGen Qinlian decoction (GGQLD) composed of Puerariae Lobatae Radix (Gegen), Scutellariae Radix (Huangqin), Coptidis Rhizoma (Huanglian), glycyrrhiza (Gancao), is the most traditional formula for diarrhoea treatment in Chinese medicine. A network pharmacology-based approach identified 130 active ingredients from GGHD and their targets were mainly related to the calcium signaling pathway, 5-HT, and gastrointestinal hormone ions which interact closely with the ENS (134). Clinical trials using GGHD in the treatment of rotavirus enteritis in children provide important insights into Chinese medicine (149). An evaluation of 34 herbs against rotavirus shows that the fruit of *Citrus aurantium* (golden mandarin) is the most effective, with the main active ingredients being neohesperidin (EC_50 =_ 25 mM) and hesperidin (EC_50 =_ 10 mM) (150).

**Table 3:** Chinese medicine and natural compounds.

Drugs	Class(es) of inhibitor(s)	Antiviral mechanism	Antiviral activity
Shenlian Baizhu Powder	Traditional Chinese Medicine	Related to TLR4/MyD88/NF-κB signaling pathway, and restoring the upregulation of inflammatory factors and downregulation of IFN-β due to rotavirus infection	NA
GeGen Qinlian Decoction	Traditional Chinese Medicine	Related to enteric nervous system	NA
the fruit of Citrus Aurantium(The active ingredient are neohesperidin and hesperidin)	Chinese herbal medicine	NA	neohesperidin (EC50 = 25 mM) and hesperidin (EC50 = 10 mM).
18β-glycyrrhetinic acid	Natural Compounds	Inhibits Fas/FasL signaling and apoptosis after RV infection.Promoting intestinal lymphocyte recruitment to the intestine	CC50 = 86.92 µg/mL, EC50 = 3.14 µg/mL, SI = 27.68
ginseng extract	Natural Compounds	Inhibits toll-like receptor 3 (TLR3)-mediated pro-inflammatory signaling pathway	NA
Macleaya cordata extracts	Herbal Medicine	Improves intestinal inflammation by inhibiting the JAK2/STAT3 pathway	NA
Resveratrol	Natural Compounds	Inhibits the expression of hsp90 mRNA and protein and blocked the early stages of the RV replication cycle	20µM
Vitamin D	Natural Compounds	Related to RIG-I signaling pathway and TBK1/IRF3 signaling pathway	1-100nM
Allium sativum	Herbal Medicine	Inhibits the ERK/MAPK signaling pathway	EC50 = 0.03 mg/mL
Pattalus mollis	Herbal Medicine	Acts before the virus enters the cells	CC50 = 27,042.10μg/mL, IC50 = 64.29μg/mL
Ursolic acid	Natural Compounds	Disturbs viroplasm synthesis and viral particle maturation	10µM
Ziyuglycoside II	Natural Compounds	Reverses the upregulation of inflammatory factors caused by RV and inhibiting TLR4/NF-κB pathway	NA
Genistein	Natural Compounds	Acts in binding and postbinding stage	30µM
Ginseng	Herbal Medicine	Interfered with the adhesion of viruses and host cells	NA
Quillaja saponaria Molina extract	Herbal Medicine	Prevents rotavirus adhesion by coated/modified host cell membranes	NA
Baicalin	Natural Compounds	Related to STAT3 and p-JNK-PDK1-AKT-SIK2 signaling pathways	NA
Alpinia katsumadai extracts	Herbal Medicine	Interacts with viral hemagglutinin protein	NA
EGCG and proanthocyanidin	Natural Compounds	Binds to viral proteins or mediating the aggregation of viral proteins	NA

Liquorice extracts show good results in *in vivo* and *in vitro* experiments with the main active component (18β-glycyrrhetinic acid, 18β-GA) inhibiting Fas/FasL signalling and apoptosis after rotavirus infection (CC50 = 86.92 µg/mL, EC50 = 3.14 µg/mL, SI = 27.68) (151, 152). Tissue cells have elevated chemokine expression after 18β-GA treatment which is indicative of B-cell recruitment to the intestine (153). This finding provides a rationale for oral drugs to promote intestinal lymphocyte enrichment and maintain the intestinal barrier.

Clinical data shows that tormentil root extract has good therapeutic effects on rotavirus *in vivo* and *in vitro* (154). Combination therapy of Sophora flavescens extract and stevioside produces a significant antiviral effect (155). Nordiacontanol is a major active ingredient of Sophora flavescens that inhibits viral replication by blocking the toll-like receptor 3 (TLR3)-mediated pro-inflammatory signaling pathway (156). Boraginaceae is a traditional European and American herb belonging to the poppy family that contains several alkaloids. A recent report shows that it has anti-rotavirus activity and improves intestinal inflammation by inhibiting the JAK2/STAT3 pathway (157). Resveratrol flavonoid is widely found in berries, and nuts, and possesses anti-rotavirus activity in cellular and animal experiments; 20 μM resveratrol inhibits viral replication in Caco-2 cells. Furthermore, it inhibits the expression of hsp90 mRNA and protein and blocks the early stages of the rotavirus replication cycle (158).

Vitamin D is a steroid hormone, and hydroxyvitamin D3 inhibits rotavirus replication in IPEC-J2 cells by activating the RIG-I signaling pathway, while vitamin D activates the autophagic pathway (159). Vitamin D inhibits rotavirus infection by targeting the TBK1/IRF3 signaling pathway *via* microRNA-155-5p, and 1-100 nM of 1α,25-dihydroxy VD3 (the hydroxylated product of vitamin D) in a dose-dependent manner (160). Garlic aqueous extract potentially inhibits PRV in MA104 cells, possibly by blocking the ERK/MAPK signaling pathway (EC_50 =_ 0.03 mg/mL) (161, 162). The sea cucumber tegument extract (*Pattalus mollis*) has a significant inhibitory effect on HRV and acts before the virus enters the cells (CC_50 =_ 27,042.10 μg/mL, IC_50 =_ 64.29 μg/mL) (163).

Ursolic acid is a natural triterpene carboxylic acid and 10 µM of this molecule inhibits rotavirus replication at all stages *in vitro*; there is a significant reduction in the number and size of virplasms at 4 h of viral infection, and viral particle maturation is affected at 15 h (164). Ziyuglycoside II is a triterpene saponin extracted from the traditional Chinese medicine, *Sanguisorba officinalis* L.; it reverses the upregulation of inflammatory factors caused by rotavirus and inhibits the TLR4/NF-κB pathway (165).

Fruits and vegetables, medicinal plants, and herbs are the main sources of polyphenols. Gopalsamy Rajiv Gandhi et.el (166) reviewed natural compounds with potential anti-rotavirus activity, of which polyphenols were the most studied, also including phenolic acids, stilbenes, tannins, pectins, terpenoids, and flavonoid glycosides, and suggested the potential of polyphenols as future anti-rotavirus drug candidates with potential anti-rotavirus activity (166). Thirty-four out of sixty flavonoid compounds exhibit anti-rotavirus activity (SI≥ 4) (167). The antiviral activity of metformin and hesperidin is validated *in vitro* (EC_50 =_ 10 mM) (168). Soy isoflavones affect all stages of viral replication, and 30 μM of genistein significantly inhibits RV infection, in the binding phase or post-binding phase (169)

Ginseng is one of the medicinal plants used to treat diarrheal diseases in traditional oriental medicine and is now used as a functional supplement which can be regularly taken to improve gastrointestinal function and prevent diarrhoea. Its main medicinal active components are saponins, polysaccharides, and flavonoids. Active ginseng polysaccharides reduce viral infection by interfering with the adhesion of viruses and host cells (170). Saponins are glycosides consisting of non-polar glycosides and varying amounts of monosaccharides, and are the main medicinal components of traditional medicinal plants such as ginseng, liquorice, and panax ginseng. The ginsenoside component of Korean red ginseng prevents diarrhoea in new-born mice (171). Roner MR (172) and Tam KI et al. (173) successively showed that *Quillaja saponaria* Molina extract blocks rotavirus adhesion *in vitro* and *in vivo*, and saponins appear to prevent rotavirus adhesion by coated/modified (wrapped/modified) host cell membranes. Saponins are water soluble and are approved as food additives. The authors suggest that oral administration of saponin with water is an affordable measure for rotavirus disruption. Baicalin is a flavonoid isolated from the root of *Radix scutellariae*. It was recently shown that the anti-rotavirus attachment and immunomodulatory effects of baicalin involve STAT3 and p-JNK-PDK1-AKT-SIK2 signalling pathways (174, 175). In addition, *Alpinia katsumadai* extracts strongly interact with viral hemagglutinin protein and prevent virus adsorption by hemagglutination inhibition assays (176). Tannins are macromolecular phenolic compounds that can form strong complexes with carbohydrates or proteins. The active substances in tannins acting against pathogens are divided into two main groups: proanthocyanidins and hydrolysable tannins (177). Proanthocyanidins are abundant in berries such as *Vaccinium macrocarpon* and *Vitis labrusca*; their juice has anti-rotavirus effects. Epigallocatechin gallate (EGCG) is a major component of tea polyphenols and its combination with proanthocyanidin-containing nutraceuticals shows synergy against rotavirus (178). The anti-rotavirus mechanism of these flavonoids is through binding to viral proteins (179) or mediating the aggregation of viral proteins (180).

## 4 Conclusion

Some progress has been made in immune protection against rotavirus. More than half of the countries in the world now introduce oral rotavirus attenuated vaccines into their national immunization programs (2); however, hundreds of thousands of rotavirus deaths continue to occur each year (181). This review focuses on recent advances in targeting different stages of the viral replication cycle, improving host immunity and gut function, and utilizing natural compounds and Chinese medicine to develop anti-rotavirus drugs. At present, oral rehydration solution, montmorillonite, probiotics, and nitazoxanide commonly used in the treatment of rotavirus enteritis in Western medicine do not achieve good results. Traditional Chinese medicine is effective in the treatment of infantile rotavirus enteritis. In recent years, there are increasing reports on natural compounds and Chinese medicine for rotavirus enteritis. Several studies (148, 151, 156, 159, 170, 171) showed that plant extracts such as ginseng and *Atractylodis macrocephalae*, *Pueraria Mirifica*, 18β-glycyrrhetinic acid, vitamin D, and Sophora flavescens extract have antiviral activity against rotavirus. Due to the anti-rotavirus effects of SBP and GHHD observed *in vitro*, we anticipated that SBP and GHHD would exert a protective effect against RV infection *via* modulation of inflammatory cytokines in the gut. In addition, SBP was first discovered in Taiping Huimin Heji Ju Fang compounded by Song Dynasty officials (1,078–1085 A.D.) and was recorded in the Pharmacopoeia of the People’s Republic of China, 2020. GHHD first recorded in the book of shanghan zabing lun (200 ~ 210 A.D.) written by Zhang Zhongjing (148, 149). These two formulas have been widely used in traditional Chinese medicine to clear away the heat and dry dampness. Clinical trials are needed to further verify their effectiveness in treating rotavirus.

## Author contributions

LJ assisted with the design of the study, participated in the acquisition of data, analysis, drafted and co-wrote the manuscript. AT participated in the acquisition of data and analysis, co-wrote the manuscript. LS assisted with the design of the study, participated in the interpretation of data, drafted and co-wrote the manuscript. YT assisted with the design of the study, participated in the interpretation of data, drafted and co-wrote the manuscript. HF assisted with the design of the study, participated in the acquisition of data, analysis and the interpretation of data, drafted and co-wrote the manuscript. All authors contributed to the article and approved the submitted version.
